# Intensity modulated radiotherapy of upper abdominal malignancies: dosimetric comparison with 3D conformal radiotherapy and acute toxicity

**DOI:** 10.1186/1748-717X-8-207

**Published:** 2013-09-05

**Authors:** Alaa Ahmad Nour, Aziz Alaradi, Adel Mohamed, Saleh Altuwaijri, Volker Rudat

**Affiliations:** 1 Department of Radiation Oncology, Saad Specialist Hospital, P.O. Box 30353, Al Khobar 31952, Saudi Arabia; 2 SAAD Research & Development Center, Saad Specialist Hospital, P.O. Box 30353, Al Khobar 31952, Saudi Arabia

## Abstract

**Background:**

The goal of this study was to assess a possible dosimetric advantage of intensity modulated radiotherapy (IMRT) of upper abdominal malignancies compared to three-dimensional conformal radiotherapy (3DCRT), and to assess the impact of IMRT on acute toxicity.

**Methods:**

Thirty-one unselected consecutive patients with upper abdominal malignancies were treated with definitive (n =16) or postoperative (n =15) IMRT. Twenty-one patients (67.7%) received concomitant chemotherapy. 3DCRT plans were generated for comparison, and analysis of variance (ANOVA) for repeated measurements was used to test for significant difference of dosimetric parameters. Acute toxicity was assessed weekly using the Common Terminology Criteria for Adverse Events (CTCAE) grading scale.

**Results:**

IMRT plans showed a small but statistically significant improvement of the conformity index compared to 3DCRT plans (difference (95% confidence interval), -0.06 (−0.109 to-0.005); p = 0.03). The homogeneity index was not significantly improved (p = 0.10). A significantly reduced high dose volume on cost of a significantly increased low dose volume was observed for the kidneys. The acute toxicity appeared to be less than commonly reported for corresponding patients treated with 3DCRT. No patient developed grade 3 or 4 non-hematological acute toxicity, and the most common grade 2 toxicity was vomiting (9.7%).

**Conclusions:**

IMRT offers the potential of a clinically relevant dosimetric advantage compared to 3DCRT in terms of a reduced acute toxicity. Further optimization of the radiotherapy technique and more clinical trials are required before IMRT is routinely used for upper abdominal malignancies.

## Introduction

Upper abdominal malignancies represent some of the most challenging cancers to treat. Although radiation therapy has an important role in the treatment of upper abdominal malignancies, delivery of adequate radiation doses is often limited by radiation sensitive normal structures in the upper abdomen. These include the kidneys, small intestine, stomach, liver, and spinal cord. Intensity modulated radiotherapy (IMRT) offers a more conformal dose distribution compared to the standard radiation technique three-dimensional conformal radiotherapy (3DCRT). IMRT may facilitate a better normal tissue sparing and dose escalation to these tumors, which has the potential to reduce toxicity and to improve local control [1].

The goal of this study was to assess a possible dosimetric advantage of IMRT compared to 3DCRT, and to assess the impact of IMRT on acute toxicity. For this purpose, IMRT and 3DCRT plans were generated for all patients and parameters of the dose distribution compared. All patients were treated with IMRT, and the acute toxicity assessed weekly using the Common Terminology Criteria for Adverse Events (CTCAE) grading scale.

### Patients and methods

IMRT and 3DCRT plans were generated for 31 unselected consecutive patients with upper abdominal malignancies who were treated between January 2010 and March 2013. The diagnoses of the upper abdominal tumors are listed in Table 1. Virtual simulation using positron emission tomography/computed tomography (PET/CT) was performed in 22 of 31 patients (71%), and conventional computed tomography (CT) simulation in nine patients (29%). All patients were treated using linac-based step and shoot IMRT. Sixteen of 31 patients (51.6%) received definitive radiotherapy, and fifteen patients (48.4%) postoperative radiotherapy. Twenty-one of 31 patients (67.7%) received concomitant radiochemotherapy using 5-FU, gemcitabine or cisplatin-based regimen, and 10 patients (32.3%) radiotherapy alone. Acute toxicity was assessed once weekly using the Common Terminology Criteria for Adverse Events (CTCAE) grading scale version 4.0.

**Table 1:** Patient and treatment characteristics

		**Total dose (Gy)**	**PTV (cm**^**3**^**)**	
**Diagnosis**	**n**	**Mean (range)**	**Mean**	**SD**
Pancreatic cancer	8	52.0 (45.0-59.4)	651	285
Cholangiocarcinoma	6	54.9 (50.4-59.4)	274	268
Ampullary and periampullary cancer	4	52.2 (50.4-54.0)	623	391
Retroperitoneal liposarcoma	3	50.4 (50.4-50.4)	2533	724
Gastro-esophageal junction cancer	3	57.3 (55.8-60.0)	362	228
Paraaortal or hepatogastric lymph node metastases	3	58.7 (56.0-60.0)	305	195
Gastric cancer	2	45.0	1241	255
Gastric malt lymphoma	1	30.0	1493	-
Adrenal gland cancer	1	55.8	1211	-

Abbreviations: *PTV* planning target volume, *SD* standard deviation.

### Treatment planning

Virtual simulation in supine position was performed either using positron emission tomography/computed tomography (Biograph 64 PET/CT, Siemens Health Care, Germany) with fluorine-18 (FDG) as tracer or non-contrast computed tomography (Somatom Sensation Open 20 Slice CT, Siemens Health Care, Germany). Both the PET/CT and CT were equipped with the same green lasers, carbon table-tops and positioning devices as used for the treatment at the linear accelerators. According to the departmental protocol a CT slice thickness of 3 mm was used for the PET/CT simulation and 5 mm for conventional CT-simulation. The isocenter was defined using the CT simulation software Coherence Dosimetrist (Siemens Medical, Germany), and target volumes (PTV and organs at risk) using the software Coherence Oncologist (Siemens Medical, Germany). The IMRT and 3DCRT plans were generated using the treatment planning system XIO 4.4 (CMS, Inc. of St. Louis, Mo, USA). Siemens Oncor Anvantgarde linear accelerators with a 160-multileaf collimator (Siemens Medical, Germany) were used for the radiotherapy. The beam energy was 6 MV for all patients. A total dose of 30.0-60.0 Gy with a single fraction dose of 1.8 Gy (26 patients) and 2.0 Gy (5 patients) was prescribed to the PTV for both the IMRT and 3DCRT plan (Table 1). For the evaluation of treatment plans tolerance doses for the organs at risk as described by the Quantitative Analysis of Normal Tissue Effects in the Clinic (QUANTEC) review [2] were used.

### Target volume definition

The target volumes were defined according to the International Commission on Radiation Units and Measurement (ICRU) Reports 50 and 62. The planning target volume (PTV) included the gross tumor volume (GTV) or preoperative GTV, draining nodal regions whenever indicated, a margin of 5 mm to 10 mm for subclinical tumor extension, a margin of 10 mm to 15 mm craniocaudally and 5 mm transversely for organ motion, and a margin of 3 mm for patients positioning accuracy. The margins for clinical extension and organ motion were adopted from the literature [3,4] and the margins for the patient positioning accuracy derived from own measurements [5]. For patients who received a PET/CT simulation the GTV was defined in cooperation with an experienced nuclear medicine physician by taking into account standardized uptake values (SUV) and visual inspection of PET/CT images. No oral contrast media, fluoroscopy or fiducial markers were used to help to define target volumes or to account for organ motion. Daily online verification using an electronic portal imaging device was performed in all patients to optimize the patient positioning accuracy.

The bowels were contoured as a whole-sum of the gastrointestinal tract tube within 2 cm above and below the craniocaudal extent of the PTV. The combined volume of the right and left kidney was used for the dose-volume histogram (DVH) analysis. The liver and the scanned part of the spinal cord were contoured entirely.

### Inverse-planned intensity modulated radiotherapy (IMRT)

The normal tissue dose volume constraints used for the IMRT plans are listed in Table 2. Tissue inhomogeneities were considered in the treatment planning optimization process, and the dose calculation algorithm used was “Superposition”. An optimization with 100 iterations was applied, followed by a semiautomatic segmentation (minimum 3 cm step size). Segments with less than ≤2 MU were expelled from the plan. A step-and-shoot technique was used with usually eight equally spaced coplanar fields. The number of segments of a typical plan was around 70 and the corresponding treatment time about 10 minutes.

**Table 2:** Dose constraints used in IMRT treatment planning

**Organ**	**Volume**	**Dose**
Ipsilateral kidney	33%	<18 Gy
	20%	<28 Gy
Contralateral kidney	30%	<6 Gy
	55%	<12 Gy
Bowel	Maximum dose	48 Gy
	15%	<45 Gy
Spinal cord	Maximum dose	40 Gy
Liver	60%	<30 Gy

### Three-dimensional conformal radiotherapy (3DCRT)

The dose was prescribed to the ICRU reference point which was usually the isocenter located in the PTV volume centroid. A three to five coplanar beam technique, multileaf collimator and virtual wedges were used for the 3DCRT. The beam angles, virtual wedges, and beam weighting were chosen to optimize coverage of the PTV, while minimizing exposure to the organs at risk kidneys, liver, bowel, stomach, and spinal cord.

Dose-volume histograms of the PTVs and organs at risk of the IMRT and 3DCRT plans were generated and dose parameters compared. The homogeneity index (HI) was defined as the fraction of the PTV with a dose between 95% and 105% of the prescribed dose (V95% - V105%). The conformity index (CI) was defined as the fraction of the PTV surrounded by the reference dose (V95%) multiplied by the fraction of the total body volume covered by the reference PTV dose ((PTV95%/PTV) × (PTV95%/V95%)) [6].

### Statistics

IMRT and 3DCRT plan parameters derived from the same patient were tested for statistically significant differences using analysis of variance (ANOVA) for repeated measurements. Parameters of the dose distribution were entered as continuous variables in the ANOVA and the timing of the radiotherapy (definitive radiotherapy versus postoperative radiotherapy) as categorical variable. Differences were considered statistically significant if the two-tailed p-value was less than or equal 0.05.

## Results

### PTV

The diagnoses of the upper abdominal malignancies examined in this study are listed in Table 1. The ANOVA for repeated measurements revealed that the conformity index of the IMRT plans was significantly better compared to the 3DCRT plans (p = 0.03) (Table 3). The homogeneity index was not significantly improved (p = 0.10). The timing of the radiotherapy (definitive RT versus postoperative RT) had no statistically significant impact on the CI or HI.

**Table 3:** Relevant plan parameters of IMRT versus 3DCRT of 31 patients with upper abdominal tumors

	**IMRT**		**3DCRT**		**Difference**			**p-value†**		
**Parameter**	**Mean**	**SD**	**Mean**	**SD**	**Mean**	**−95CI**	**+95CI**	**Timing of RT**	**Plan**	Plan*Timing of RT
PTV										
HI	0.97	0.03	0.96	0.04	−0.01	−0.027	0.003	0.46	0.10	0.40
CI	0.71	0.20	0.65	0.23	−0.06	−0.109	−0.005	0.17	0.03	0.22
Combined kidneys										
Mean dose (cGy)	1428	640	1322	656	−106	−256	44	0.01	0.16	0.46
Maximum Dose (cGy)	3946	1418	4272	1130	325	58	593	0.06	0.02	0.23
V12	59.7	31.9	41.3	23.03	−18.4	−28.6	−8.3	0.13	<0.01	0.75
V20	26.8	19.4	22.3	17.7	−4.5	−8.63	−0.41	0.11	0.04	0.89
V28	10.8	12.395	13.9	14.0	3.1	0.4	5.9	0.15	0.03	0.41
Liver										
Mean dose (cGy)	1593	817	1427	890	−165	−261	−69	0.39	<0.01	0.34
Maximum Dose (cGy)	4924	945	4592	1377	−332	−723	58	0.76	0.10	0.67
Small bowel										
Mean dose (cGy)	4394	1686	4237	1800	−157	−556	242	0.27	0.39	0.15
V15	62.2	22.5	54.9	20.9	−7.3	−1.0	−4.6	0.67	<0.01	0.19
Spinal cord										
Maximum Dose (cGy)	2940	1003	2999	1208	59	−269	386	0.83	0.72	0.89

IMRT = Reversed planned intensity modulated radiotherapy; 3DCRT = Three-dimensional planned conformal radiotherapy; CI = 95% confidence interval: Timing of RT = Definitive radiotherapy versus postoperative radiotherapy; † = ANOVA with repeated measurements; Vx = Percentage of tissue volume encompassed by the x cGy isodose line; Plan = IMRT versus 3DCRT; Plan*Timing of RT = Interaction between plan and timing of RT.

### Organs at risk

For the combined kidneys, IMRT showed a significantly reduced high dose volume (maximum dose and V28) on cost of a significantly increased low dose volume (V20 and V12) (Table 3). No clinically relevant differences were found for the liver, bowel and spinal cord. There was no significant interaction between the timing of the radiotherapy (definitive versus postoperative radiotherapy) and the observed differences of the dose distribution between IMRT and 3DCRT plans (Table 3).

### Acute toxicity

A striking clinical observation was the low acute toxicity of patients treated with IMRT. No patient developed grade 3 or 4 non-hematological acute toxicity, and the most common grade 2 toxicity was vomiting (9.7%) (Table 4). The acute toxicity observed in our study appears to be less than expected from patients treated with 3DCRT. Reported non-hematological acute toxicities grade 3 or higher of patients with pancreatic cancer treated with definitive or postoperative radiochemotherapy ranged from 10% to 19% [7-9]. However, comparison with historical data has to be interpreted with great caution.

**Table 4:** Acute reactions of patients with upper abdominal tumors treated with radiochemotherapy (n = 21) or radiotherapy alone (n = 10)

	**Grade CTCAE v4**			
**Adverse event**	**0**	**1**	**2**	**3–4**
Nausea	51.6%	45.2%	3.2%	0%
Vomiting	80.6%	9.7%	9.7%	0%
Diarrhea	80.6%	19.4%	0.0%	0%
Weight loss	67.6%	29.0%	3.2%	0%

Abbreviation: *CTCAE v4* common terminology criteria for adverse events version 4.0.

## Discussion

Our dosimetric comparison showed a small but statistically significant improvement of the conformity index of IMRT compared to 3DCRT plans for tumors located in the upper abdomen. In addition, IMRT significantly reduced the high-dose volume on cost of an increased low-dose volume of the kidneys. A striking clinical observation of our study was the generally low acute toxicity of patients treated with IMRT. It can be speculated that the low acute toxicity observed may be due to the improved conformity index indicating a reduced high dose volume around the tumor, and that in the upper abdomen relatively small changes of the dose distribution may have a significant effect on the acute toxicity.

In agreement with our study, several other reports described a more favourite dose distribution of IMRT compared to 3DCRT for malignancies of the upper abdomen. Poppe et al. compared helical intensity-modulated radiotherapy (HIMRT), linac-based IMRT and 3DCRT in eight resected and eight unresected pancreatic cancer patients [4]. Both HIMRT and IMRT offered a statistically significant improvement over 3DCRT in lowering the dose to liver, stomach, and bowel. The results were similar for both resected and unresected patients. Ringash et al. assessed the potential advantage of IMRT over 3DCRT for postoperative adjuvant radiotherapy in patients with gastric carcinoma [10]. In 17 out of 19 (89%) patients the IMRT plan was preferred over the corresponding 3DCRT plan. The target coverage was improved, while spinal cord, liver, kidney and heart dose was reduced in 69% to 74% of the patients. Murthi et al. assessed the potential advantage of IMRT over 3DCRT planning in postoperative adjuvant radiotherapy for 15 patients with gastric carcinoma [11]. The IMRT plans achieved statistically significant better target coverage with higher conformity index value compared to 3DCRT plans. The doses to the liver and bowel reduced significantly with IMRT plans compared to 3DCRT plans. For all organs at risk the percentage of volumes receiving more than their tolerance doses were reduced with the IMRT plans. Wieland et al. compared IMRT, 3DCRT and anterior-posterior opposed (AP-PA) beam arrangement in 15 gastric cancer patients to assess the potential of IMRT to reduce radiation toxicity [12]. On average, median dose to the right kidney was the same for the conventional box technique and IMRT but lower for the AP-PA technique. In three patients, kidney dose might have been ablative for both kidneys with both the AP-PA technique and the box technique, whereas it was acceptable with IMRT. The authors concluded that IMRT can deliver efficient doses to target volumes while delivering dose to the kidneys in a fashion that is different from a conventional technique and is clearly advantageous in a small number of patients. A plan comparison study of 14 patients with gastric cancer by Alani et al. revealed a satisfactory coverage of the PTV by the 95% isodose envelope using either IMRT or 3DCRT [13]. However, IMRT was only marginally better than 3DCRT at protecting the spine and kidneys from radiation. The authors concluded that IMRT confers only a marginal benefit in the adjuvant treatment of gastric cancer and should be used only in the small subset of patients with risk factors for kidney disease or those with a preexisting nephropathy. In a dosimetric study involving 10 patients with cancer of the distal esophagus and gastrointestinal junction, target heterogeneity was improved in eight of ten patients by IMRT compared to 3DCRT, and the conformity index improved with the number of beams used for IMRT (4-beam versus 7-beam versus 9-beam IMRT) [14].

The impact of IMRT on acute gastrointestinal toxicity was assessed in 46 patients with pancreatic and ampullary cancer by Yovino et al. [15]. Compared to patients treated with 3DCRT in the ROTG 97–04 trial the incidence of grade 3–4 nausea and vomiting (0% versus 11%, p = 0.024) and diarrhea (3% versus 18%, p = 0.017) was significantly reduced. Similar observations of reduced gastrointestinal acute toxicity by IMRT compared to published data of patients treated with 3DCRT have been reported by several other study groups [16-19].

Our study suggests that IMRT of malignancies of the upper abdomen compared to 3DCRT offers a dosimetric advantage and reduced acute toxicity. It should be noted that we compared IMRT with 3-field or 5-field 3DCRT plans and that more sophisticated 3DCRT plans may yield more optimized dose distributions in selected patients. Furthermore, it should be stressed that the more conformal dose distribution of IMRT may increase the risk of geometrical miss and consequently the likelihood of local failure.

Advances in diagnostic imaging, image-guided radiotherapy as well as patient immobilization and organ motion management throughout treatment planning and treatment are required to optimize the radiotherapy of upper abdominal malignancies [1,20-24]. Furthermore, a better understanding of the partial volume tolerances of the normal tissues is required to optimize the radiotherapy treatment planning and to select the plan with the best biologically effective dose distribution [1]. However, a recent analysis of patterns of failure among 71 patients treated with adjuvant IMRT for pancreas cancer suggested that IMRT was not associated with an increase in local recurrences [25].

In conclusion, IMRT of upper abdominal malignancies offers the potential of a clinically relevant advantage of the dose distribution compared to 3DCRT in terms of a reduced acute toxicity. Further optimization of the radiotherapy technique and more clinical trials are required before IMRT is routinely used for upper abdominal malignancies.

## Abbreviations

CI: Conformity index; GTV: Gross tumor volume; HI: Homogeneity index; IMRT: Intensity modulated radiotherapy using inverse treatment planning; PTV: Planning target volume; VX%: Percentage of tissue encompassed by the X% isodose line, representing the volume of tissue that receives at least 95% of the prescribed dose; 3DCRT: Three-dimensionally planned conformal radiotherapy.

## Competing interests

The authors declare that they have no competing interests.

## Authors’ contributions

AN participated in the design and coordination of the study, in the treatment planning, and helped to draft the manuscript. AA and AM carried out the dose calculation and helped to draft the manuscript. SA participated in the coordination and helped to draft the manuscript. VR conceived of the study, participated in the design, coordination, and treatment planning, performed the statistical analysis, and helped to draft the manuscript. All authors read and approved the final manuscript.
